# Determinants and Regression Equations for the Calculation of *z* Scores of Left Ventricular Tissue Doppler Longitudinal Indexes in a Healthy Italian Pediatric Population

**DOI:** 10.1155/2015/380729

**Published:** 2015-11-22

**Authors:** Veronica Fibbi, Piercarlo Ballo, Silvia Favilli, Gaia Spaziani, Giovanni B. Calabri, Iva Pollini, Alfredo Zuppiroli, Enrico Chiappa

**Affiliations:** ^1^Cardiology Unit, S. Maria Annunziata Hospital, Florence 50012, Italy; ^2^Department of Pediatric Cardiology, Meyer Hospital, Florence 50139, Italy; ^3^Regional Health Agency of Tuscany, Florence 50141, Italy

## Abstract

*Aim.* We investigated the predictors of tissue Doppler left ventricular (LV) longitudinal indexes in a healthy Italian pediatric population and established normative data and regression equations for the calculation of *z* scores.* Methods and Results.* A total of 369 healthy subjects aged 1–17 years (age of 6.4 ± 1.1 years, 49.1% female) underwent echocardiography. LV peak longitudinal velocity at systole (*s* ^'^), early diastole (*e* ^'^), and late diastole (*a* ^'^) was determined by tissue Doppler. The ratio of peak early diastolic LV filling velocity to *e* ^'^ was calculated. Age was the only independent determinant of *s* ^'^ (*β* = 0.491, *p* < 0.0001) and the strongest determinant of *e* ^'^ (*β* = 0.334, *p* < 0.0001) and *E*/*e* ^'^ (*β* = −0.369, *p* < 0.0001). Heart rate was the main determinant of *a* ^'^ (*β* = 0.265, *p* < 0.0001). Male gender showed no effects except for a weak association with lateral *s* ^'^, suggesting no need of gender-specific reference ranges. Age-specific reference ranges, regression equations, and scatterplots for the calculation of *z* scores were determined for each index.* Conclusion.* In a pediatric Italian population, age was the strongest determinant of LV longitudinal dynamics. The availability of age-specific normality data for the calculation of *z* scores may allow for correctly detecting LV dysfunction in pediatric pathological populations.

## 1. Introduction

Tissue Doppler (TD) imaging is an established echocardiographic technique that provides reproducible, sensitive, and easy-to-calculate indexes of longitudinal left ventricular (LV) function [1, 2]. Though the majority of TD studies have been performed in adults, TD has also been widely used in pediatric populations as a tool to detect early LV longitudinal dysfunction in several pathophysiological conditions, in most cases by comparing patients with matched controls [3–7]. However, few data exist regarding the determinants and the reference ranges of LV longitudinal indexes in the pediatric age [8–10]. From a clinical point of view, investigating the determinants of longitudinal LV velocities in a healthy pediatric population might be helpful to better understand the mechanisms underlying LV failure in patients with congenital heart disease. Also, obtaining normative data for LV longitudinal velocities, particularly referred to as the possibility of allowing calculation of *z* scores, may be important to provide a tool to rapidly identify early LV dysfunction in daily practice.

The aim of this study was to explore the determinants of LV longitudinal indexes in a healthy Italian pediatric population and to provide normative data obtained in a healthy Italian pediatric population. Regression equations for each index were obtained, to allow the calculation of corresponding *z* scores.

## 2. Materials and Methods

### 2.1. Study Population

Consecutive healthy subjects aged 1 to 17 years, visited at the Department of Pediatric Cardiology of Anna Meyer Children Hospital, Florence, Italy, over a 9-month enrolment period, were prospectively enrolled in this study. Subjects were deemed as normal if they had unremarkable clinical history, normal findings at clinical examination, ECG, standard echocardiography, and no family history of genetic cardiac disease (e.g., Marfan's syndrome or cardiomyopathy) and if they were not on pharmacological agents. Patients with technically inadequate image quality were excluded. Main reasons for referral were cardiovascular assessment for nonagonistic sport activities (*n* = 209), innocent cardiac murmur (*n* = 70), vasovagal lipothymia (*n* = 27), fever (*n* = 23), atypical chest pain (*n* = 22), and family history of cardiac disease (*n* = 18). The study protocol agreed with the 1964 Helsinki declaration and successive emendations and was approved by the local Ethical Committee.

### 2.2. Echocardiography

Studies were performed with use of high-quality commercially available ultrasound systems (IE 33, Philips Medical Systems, Andover, MA). LV dimensions and LV mass were measured using M-mode imaging from the parasternal long-axis view, in accordance with current ASE/EACVI recommendations [11]. End-diastolic and end-systolic LV volumes were calculated from apical views using the biplane modified Simpson's rule. Left atrial volume at end systole was measured from apical views using the biplane method of discs. LV mass, LV volumes, and left atrial volume were considered in the analysis after indexation to body surface area [12, 13]. Pulsed wave Doppler analysis of LV inflow was performed in the apical 4-chamber view, by placing the cursor at the level of the mitral leaflet tips. Early to late peak filling velocity ratio and deceleration time were measured. Pulsed TD of LV mitral annulus was performed in the apical 4-chamber views by placing the sample volume at the septal and lateral annular sites, in accordance with current guidelines [14]. To optimize quality of pulsed TD, filters and gains were adjusted at the minimal optimal level allowing the best signal-to-noise ratio. The peaks of myocardial systolic (*s*′), early diastolic (*e*′), and late diastolic (*a*′) waves were determined. Average *s*′, *e*′, and *a*′ were obtained as the mean of septal and lateral values. Septal, lateral, and average *E*/*e*′ ratio were also calculated. Right ventricular systolic function was assessed by measuring tricuspid annulus plane systolic excursion by M-mode and peak systolic velocity of tricuspid annulus by pulsed TD. All measurements were taken by averaging values obtained in three consecutive cycles.

Reproducibility was assessed in a subset of 40 randomly selected subjects. For intraobserver analysis, one investigator reviewed echocardiographic images and measured septal and lateral TD velocities, with at least one month interval between measurements. For interobserver analysis, two experienced investigators independently reviewed images. Intraobserver variability coefficients for *s*′, *e*′, *a*′, and *E*/*e*′ were as follows: average, 4.0%, 3.2%, 4.4%, and 3.1%; septal, 4.2%, 3.7%, 4.5%, and 4.0%; and lateral, 4.4%, 3.9%, 4.6%, and 4.1%. Corresponding intraclass correlation coefficients were as follows: average, 0.95, 0.96, 0.97, and 0.96; septal, 0.89, 0.92, 0.93, and 0.96; lateral, 0.97, 0.97, 0.95, and 0.96; and *p* < 0.0001 for all. Interobserver variability coefficients were as follows: average, 4.3%, 3.6%, 4.8%, and 3.3%; septal, 4.8%, 4.3%, 5.0%, and 4.5%; and lateral, 4.7%, 4.1%, 5.8%, and 4.2%. Corresponding intraclass correlation coefficients were as follows: average, 0.94, 0.95, 0.96, and 0.93; septal, 0.84, 0.90, 0.92, and 0.95; lateral, 0.96, 0.97, 0.94, and 0.96; and *p* < 0.0001 for all.

### 2.3. Statistical Analysis

Data are presented as mean ± SD or number (percentages). Correlations were expressed using Pearson's coefficients. Gender comparison was performed using the Student *t*-test for unpaired data. For the determination of age-specific reference ranges, the following age intervals were predefined: 1-2 years, 3-4 years, 5-6 years, 7-8 years, 9–11 years, and 12–16 years. Multivariable regression analysis was used to explore the independent determinants of TD indexes. Models were obtained by testing all variables with univariable *p* < 0.10 in a stepwise multivariable analysis. A *F*-to-remove of >0.10 and *F*-to-enter of <0.05 were used as criteria for the selection of variables in the stepwise procedure. Collinearity diagnostics were performed to explore model stability, setting a cut-off of <0.20 for tolerance for the identification of significant multicollinearity. In case of multivariable models with evidence of collinearity problems, the variable with the highest variance inflation factor was removed from the model. This procedure was iterated until a stable model was obtained. The goodness-of-fit was expressed using the adjusted *R* ^2^.

For the generation of regression equations for LV longitudinal indexes, the following procedure was used. First, to account for heterogeneous variances across the range of age, a natural logarithmic transformation was performed for each index to stabilize variances. Second, nonlinear regression analysis was performed by testing different polynomial equations and choosing the best fitting model according to the Akaike Information Criterion. For all LV longitudinal indexes, a third-grade polynomial regression was found to provide the best fitting. A cubic regression model of the form *Y* = *b*_3_ Age^3^ + *b*_2_ Age^2^ + *b*_1_ Age + *b*_0_ was then obtained for each index, where *Y* represents the expected value of logarithmic-transformed longitudinal index according to age. Third, values were converted to the original units by exponentiating Log *Y* and then allowing calculation of the expected value of the echocardiographic index according to age. In addition to the curve showing the expected values, other 6 curves corresponding to the *z* scores ±1, ±2, and ±3 were generated for each index by adding or subtracting the corresponding mean square error (MSE) to the regression equation. The significance level for all analyses was set at 0.05. All tests were two-tailed. Analyses were performed using the SPSS (Statistical Package for Social Sciences, Chicago, Illinois) for Windows, release 15.0.

## 3. Results

### 3.1. General Characteristics

A total of 393 subjects met the inclusion criteria during the period of study. Adequate measurement of TD velocities was not possible in 24 subjects (in one case because of poor acoustic windows secondary to pectus excavatum, in the other cases because of uncontrollable crying in subjects aged <4 years). Main characteristics of the remaining 369 subjects are shown in Table 1.

**Figure 1:**
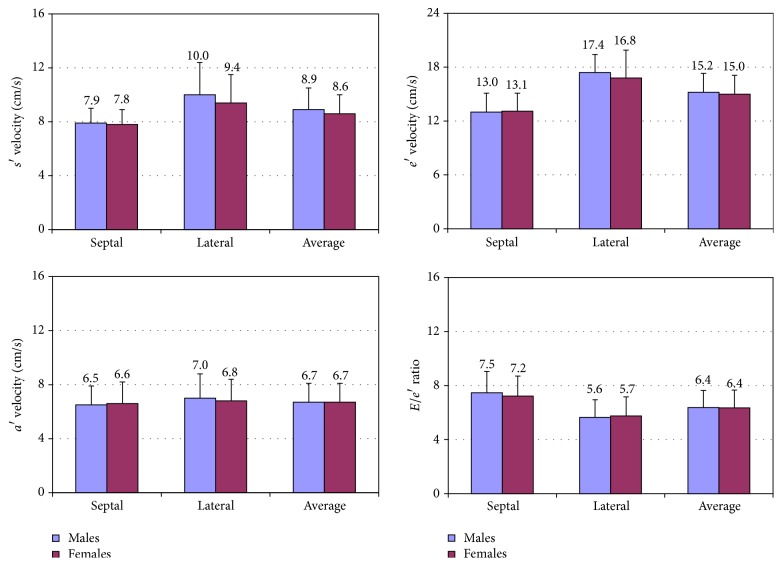
Comparison of left ventricular (LV) longitudinal indexes between male and female subjects in the study population. *a*′ is peak late diastolic LV longitudinal velocity; *e*′ is peak early diastolic LV longitudinal velocity; *E* is peak early diastolic left ventricular filling velocity; *s*′ is peak systolic LV longitudinal velocity.

Univariable correlations of LV longitudinal indexes with clinical and echocardiographic variables are shown in Table 2. Most indexes showed significant relationships with age, body surface area, systolic and diastolic blood pressure, heart rate, LV end-diastolic and end-systolic volumes, LV mass, left atrial volume, LV inflow indexes, and measures of right ventricular systolic function. However, the relations with LV volumes and left atrial volume were no longer significant or become considerably weaker when indexed volumes were considered. The strongest associations of *s*′, *e*′, and *E*/*e*′ were observed with age, whereas the strongest association of *a*′ was observed with heart rate. Gender comparison (Figure 1) showed no differences in average *e*′, *a*′, and *E*/*e*′ (*p* = NS for all), whereas slightly higher average *s*′ velocities were found in male as compared to female subjects (*p* = 0.017). Reference ranges according to predefined age classes are shown in Table 3.

### 3.2. Regression Analysis for Calculation of *z* Scores

The results of regression analysis are shown in Table 4. For each LV longitudinal index, coefficients for the cubic, quadratic, and linear terms are provided, together with the intercept, the MSE, and the *R* ^2^ value as an overall measure of model goodness-of-fit. The table allows an exact calculation of the *z* score for any observed value *y* of a LV longitudinal index in a patient with given age. This can be achieved by inserting patient's age in the corresponding equation, so that the expected value *Y* of the logarithmic-transformed longitudinal index is obtained. Then, the *z* score can be calculated using the standard formula [Log(observed *y*) − *Y*]/MSE. For example, in a 12-year-old patient with an *e*′ of 12.6 cm/s, the expected Log *e*′ value is given by (2.6 · 10^−4^) · 12^3^ − (8.4 · 10^−3^) · 12^2^ + 0.088 · 12 + 2.45 = 2.746 (which corresponds to an expected *e*′ of *e* ^2.746^ = 15.6 cm/s). The *z* score is obtained as [Log(12.6) − 2.746]/0.141 = −1.51.

**Figure 2:**
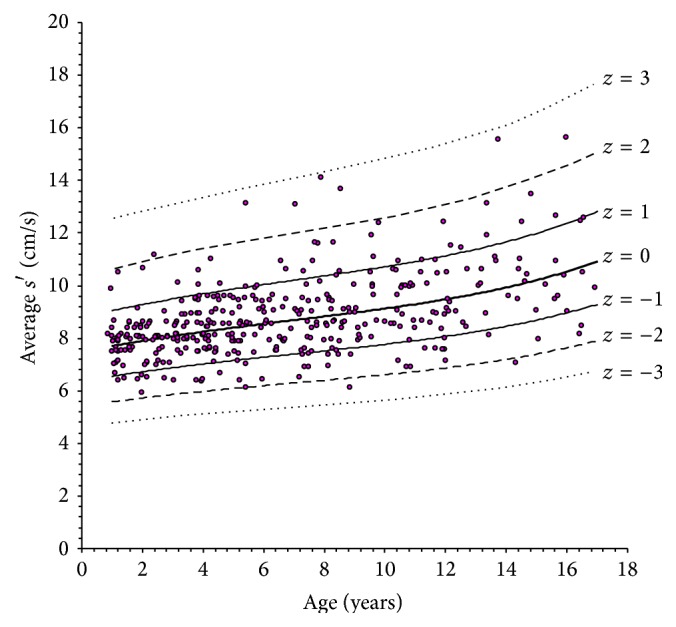
Scatterplot of peak systolic left ventricular longitudinal velocity (*s*′) versus age. In this figure and in the successive ones, the solid line represents the estimated regression equation (indicated as *z* = 0). The other lines represent the ±1, ±2, and ±1 *z* values above and below the regression curve.

**Figure 3 fig3:**
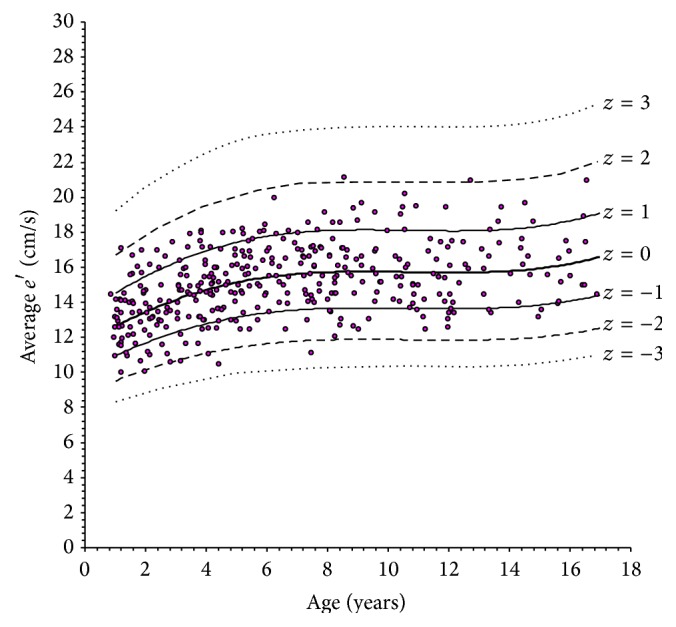
Scatterplot of peak early diastolic left ventricular longitudinal velocity (*e*′) versus age.

**Figure 4 fig4:**
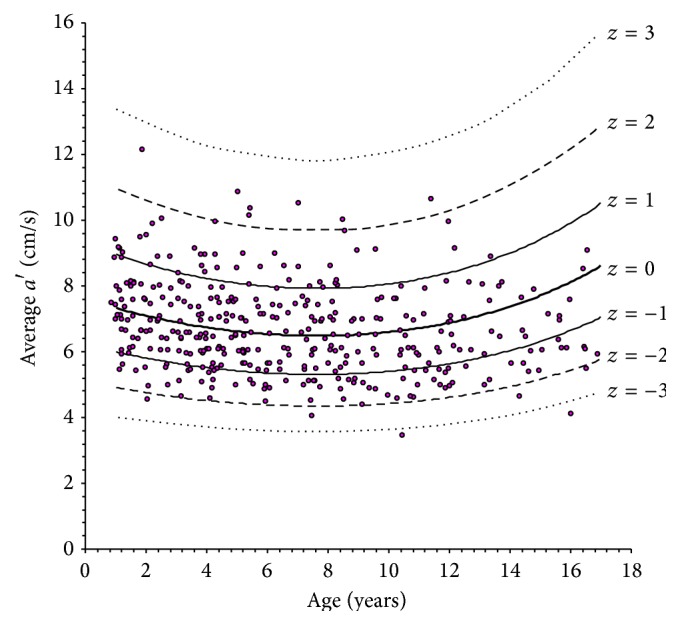
Scatterplot of peak late diastolic left ventricular longitudinal velocity (*a*′) versus age.

**Figure 5:**
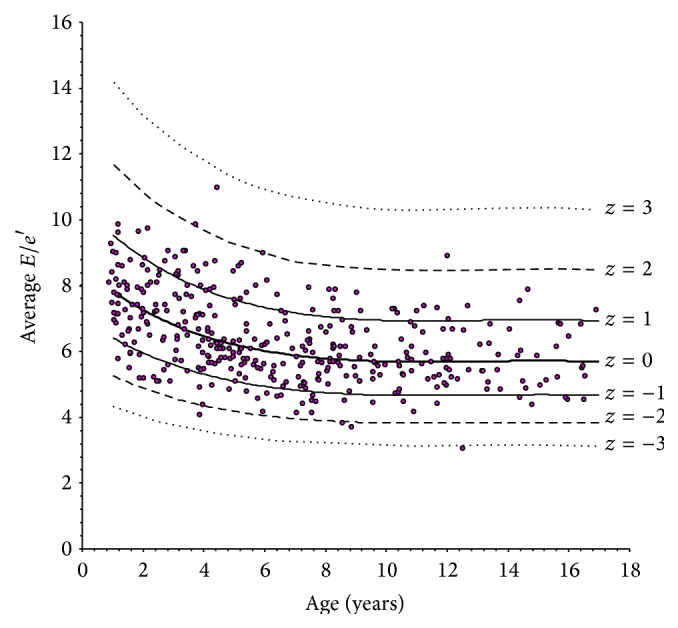
Scatterplot of the ratio between peak early diastolic left ventricular filling velocity (*E*) and peak early diastolic longitudinal velocity (*e*′) versus age.

The scatterplots of LV longitudinal indexes obtained against age are shown in Figures 2 [Fig fig3] [Fig fig4]–5. In each plot, the thick solid line is the regression equation, whereas the other 6 curves represent the values corresponding to ±1, ±2, and ±3 *z* scores. These figures allow an alternative, less precise but easier and more rapid estimation of *z* scores, which can be useful for quick application in daily practice. For any observed value *y* of a LV longitudinal index in a patient with given age, it is sufficient to ideally draw a vertical line corresponding to patient's age and a horizontal line corresponding to *y* and to look at where the two lines cross. Considering the example above, it is easy to observe in Figure 2 that the crossing point corresponding to an *e*′ value of 12.6 cm/s in a 12-year subject falls approximately midway between the *z* = −1 and *z* = −2 curves, which directly leads to an estimated *z* score of around −1.5.

### 3.3. Determinants of LV Longitudinal Indexes

Multivariable analysis (Table 5) confirmed that age was the main independent determinant of *s*′, *e*′, and *E*/*e*′ ratio, whereas heart rate was the strongest independent determinant of *a*′. Age was the only predictor of *s*′ and accounted for 76.6% and 96.7% of the total variability expressed by the *e*′ and *E*/*e*′ models, respectively. Heart rate accounted for 73.1% of the total variability expressed by the *a*′ model. Notably, as a result of strong interrelations with age, most of other variables associated with LV longitudinal indexes at univariable analysis such as body surface area, heart rate, and measures of cardiac growth were removed because of violation of collinearity criteria. Collinearity diagnostic confirmed that forcing any of them into the models yielded unacceptable levels of variance inflation factor and tolerance.

## 4. Discussion

### 4.1. Main Findings

Accurate assessment of LV function is a key aspect in the management of children with several types of congenital and acquired cardiac disease. In this study, we investigated the independent determinants of indexes of LV longitudinal function in a normal Italian pediatric population and sought to provide normative data for the calculation of *z* scores. We found that age, but not gender, was the strongest determinant of most indexes. We then derived age-specific reference ranges and regression equations for the calculation of *z* scores for each index.

### 4.2. Determinants of LV Longitudinal Indexes

To our knowledge, only two studies previously explored the determinants of longitudinal LV function in healthy pediatric subjects. In a retrospective analysis of 325 healthy American children, Eidem et al. [8] found that unindexed LV end-diastolic diameter and age were the strongest predictors of mitral annulus systolic and early diastolic velocities and that weaker associations existed with heart rate. Also, unindexed LV end-diastolic diameter and LV mass were the only determinants of the *E*/*e*′ ratio. In another study on 242 healthy Chinese children, Liu et al. [9] found that LV end-diastolic diameter was the most important predictor of all LV longitudinal indexes except for *a*′ and that weaker independent relationships existed with age, body surface area, and heart rate. Our results confirm the important independent role of age as a major determinant of systolic and early diastolic mitral annulus velocities in the pediatric age but also showed that the associations with body surface area, heart rate, and measures of cardiac growth such as LV dimensions and LV mass were no longer significant in multivariable models. We also found that age was the strongest determinant of the *E*/*e*′ ratio. Several factors could explain these discrepancies across studies. First, the different geographic locations where the studies were performed could have played a role. Second, the use of LV volumes in our study, instead of LV diameters used in previous analyses, might have provided more reliable measures of cardiac growth. Third, the use of unindexed values of LV dimensions and mass in previous studies may have favoured the selection of these variables in multivariable models. Accordingly, in our population, indexing LV volumes, LV mass, and left atrial volume dramatically reduced the strength of the associations with LV longitudinal indexes. Fourth, the restrictive collinearity criteria adopted in our analysis may have contributed to removal of highly interrelated variables such as body surface area, heart rate, and measures of cardiac growth.

### 4.3. Normative Data and Regression Equations

Few data also exist about the normal ranges of LV longitudinal indexes in children. After early studies based on relatively small sample sizes and old TD software [15, 16], the above-mentioned study by Eidem et al. provided reference ranges for LV longitudinal indexes in a large sample of normal American children [8]. More recently, van der Hulst et al. reported reference ranges of LV longitudinal indexes in 123 healthy Dutch children [10]. Our data may add to these findings by providing age-specific normality ranges in an Italian pediatric population and reporting regression equations and plots for the calculation of *z* score. In this regard, because of the lack of significant effect of gender on LV longitudinal indexes, we avoided calculation of sex-specific reference ranges. In addition, to take into account the expectable reduction of cardiac growth rate with increasing age, we predefined relatively narrow age classes for subjects in the first years of life compared to those for older children and teenagers. This allowed us to obtain distinct normative data for narrower age classes in comparison with previous studies, encompassing two- or three-year ranges until 11 years of age.

### 4.4. Clinical Implications and Study Limitations

The findings of this study provide age-specific normality ranges and two different approaches for the calculation of *z* scores for LV longitudinal indexes, which could be used to estimate deviation from normality in clinical practice. The availability of adequate normative data for LV longitudinal indexes in children is important to allow correct identification of abnormal LV longitudinal function in pathological populations, particularly in those with congenital heart disease [17]. LV longitudinal systolic indexes are well known to be early and sensitive markers of systolic dysfunction and can be used to detect subtle contractile fiber impairment even in subjects with preserved LV ejection fraction and normal indexes of LV circumferential shortening [18]. On the other hand, subtle LV longitudinal systolic dysfunction in children has been shown to predict adverse outcome in various congenital and acquired pathophysiological conditions [19–23]. Moreover, knowledge of normal values for diastolic longitudinal velocities in children is crucial to allow correct identification of LV diastolic impairment, which is a key feature of many congenital heart diseases [24–26].

Some limitations should be considered in this study. A larger sample size could have allowed us to obtain even narrower age classes. All measurements were taken using equipment from one vendor. Since previous evidence showed that different vendors cannot be used interchangeably for exploration of myocardial velocities and strains [27–29], caution is required in generalizing our findings. Speckle tracking is known to be a technique that can overcome most of the limitations of TD, particularly angle-dependency and sensitivity to passive motion [30, 31]. Further studies aimed at defining normative data for LV longitudinal strain measures are warranted.

## Figures and Tables

**Table 1:** Main clinical and echocardiographic characteristics of the study population.

Age (years)	6.4 ± 1.1
Female gender (%)	181 (49.1%)
Weight (kg)	26.0 ± 14.6
Height (m)	117.6 ± 27.2
Body mass index (kg/m^2^)	17.5 ± 3.6
Body surface area (m^2^)	0.91 ± 0.36
Systolic blood pressure (mmHg)	99.0 ± 9.3
Diastolic blood pressure (mmHg)	61.9 ± 6.7
Heart rate (bpm)	94.0 ± 20.1
Indexed LV end-diastolic volume (mL/m^2^)	53.4 ± 13.2
Indexed LV end-systolic volume (mL/m^2^)	19.3 ± 6.0
Ejection fraction (%)	63.9 ± 4.7
Indexed LV mass (g/m^2^)	62.0 ± 13.8
*E* wave (cm/s)	94.2 ± 13.8
*A* wave (cm/s)	51.0 ± 15.4
*E*/*A* ratio	2.0 ± 0.6
Deceleration time (ms)	141.1 ± 33.6
Indexed LA volume (mL/m^2^)	19.4 ± 5.5
Septal *s*′ (cm/s)	7.9 ± 1.1
Septal *e*′ (cm/s)	13.1 ± 2.0
Septal *a*′ (cm/s)	6.5 ± 1.5
Septal *e*′/*a*′ ratio	2.1 ± 0.6
Septal *E*/*e*′ ratio	7.3 ± 1.6
Lateral *s*′ (cm/s)	9.7 ± 2.3
Lateral *e*′ (cm/s)	17.1 ± 3.1
Lateral *a*′ (cm/s)	6.9 ± 1.7
Lateral *e*′/*a*′ ratio	2.6 ± 0.8
Lateral *E*/*e*′ ratio	5.7 ± 1.4
Average *s*′ (cm/s)	8.8 ± 1.5
Average *e*′ (cm/s)	15.1 ± 2.1
Average *a*′ (cm/s)	6.7 ± 1.4
Average *e*′/*a*′ ratio	2.3 ± 0.6
Average *E*/*e*′ ratio	6.3 ± 1.3

*A*: peak late diastolic transmitral flow velocity; *a*′: peak late diastolic mitral annulus velocity; *E*: peak early diastolic transmitral flow velocity; *e*′: peak early diastolic mitral annulus velocity; LV: left ventricular; *s*′: peak systolic mitral annulus velocity.

**Table 2:** Univariable relationships of tissue Doppler indexes with clinical and echocardiographic variables in the study population. Values were computed by considering the average of velocities taken at the septal and lateral site.

	Average *s*′		Average *e*′		Average *a*′		Average *E*/*e*′	
	*R*	*p*	*R*	*p*	*R*	*p*	*R*	*p*
Age	0.49	<0.0001	0.40	<0.0001	−0.20	<0.0001	−0.43	<0.0001
Body mass index	0.15	0.0044	0.04	0.50	0.01	0.89	−0.05	0.37
Body surface area	0.49	<0.0001	0.37	<0.0001	−0.20	<0.0001	−0.41	<0.0001
Systolic blood pressure	0.38	<0.0001	0.21	<0.0001	0.04	0.47	−0.23	<0.0001
Diastolic blood pressure	0.29	<0.0001	0.19	0.0003	−0.03	0.55	−0.26	<0.0001
Heart rate	−0.17	0.0014	−0.31	<0.0001	0.35	<0.0001	0.35	<0.0001
LV end-diastolic volume	0.43	<0.0001	0.34	<0.0001	−0.19	0.0002	−0.33	<0.0001
Indexed LV end-diastolic volume	0.11	0.0030	0.13	0.15	−0.08	0.11	−0.05	0.31
LV end-systolic volume	0.37	<0.0001	0.28	<0.0001	0.28	<0.0001	−0.32	<0.0001
Indexed LV end-systolic volume	0.04	0.40	0.06	0.22	−0.07	0.16	−0.05	0.37
LV mass	0.48	<0.0001	0.33	<0.0001	−0.14	0.0075	−0.33	<0.0001
Indexed LV mass	0.27	<0.0001	0.14	0.0056	−0.03	0.57	−0.10	0.066
Ejection fraction	0.13	0.011	0.11	0.029	0.04	0.45	−0.04	0.47
Left atrial volume	0.41	<0.0001	0.34	<0.0001	−0.13	0.011	−0.32	<0.0001
Indexed left atrial volume	0.04	0.45	0.08	0.13	0.02	0.65	−0.03	0.61
Mitral *E*/*A* ratio	0.03	0.56	0.28	<0.0001	−0.30	<0.0001	−0.07	0.15
Deceleration time	0.23	<0.0001	0.22	<0.0001	−0.14	0.010	−0.19	0.0004
TAPSE	0.31	<0.0001	0.23	<0.0001	−0.01	0.77	−0.10	0.047
Tricuspid *s*′	0.36	<0.0001	0.19	0.0003	0.11	0.036	−0.06	0.24

*A*: peak late diastolic transmitral flow velocity; *a*′: peak late diastolic mitral annulus velocity; *E*: peak early diastolic transmitral flow velocity; *e*′: peak early diastolic mitral annulus velocity; LV: left ventricular; *s*′: peak systolic mitral annulus velocity; TAPSE: tricuspid annulus plane systolic excursion.

**Table 3:** Reference ranges of left ventricular longitudinal indexes according to age class, expressed as mean ± standard deviation.

	1-2 y (*n* = 73)	3-4 y (*n* = 77)	5-6 y (*n* = 53)	7-8 y (*n* = 61)	9–11 y (*n* = 49)	12–16 y (*n* = 56)
Septal *s*′ (cm/s)	7.5 ± 0.9	7.8 ± 1.0	7.5 ± 1.0	7.9 ± 1.0	8.0 ± 0.9	8.6 ± 1.4
Septal *e*′ (cm/s)	12.1 ± 1.9	12.9 ± 1.7	13.1 ± 2.2	13.4 ± 2.1	13.8 ± 1.7	13.8 ± 2.3
Septal *a*′ (cm/s)	7.2 ± 1.6	6.6 ± 1.3	6.4 ± 1.6	6.3 ± 1.4	6.0 ± 1.1	6.4 ± 1.6
Septal *e*′/*a*′ ratio	1.8 ± 0.5	2.0 ± 0.5	2.2 ± 0.6	2.2 ± 0.6	2.4 ± 0.6	2.3 ± 0.5
Septal *E*/*e*′ ratio	8.1 ± 1.4	7.7 ± 1.4	7.5 ± 1.6	6.9 ± 1.5	6.8 ± 1.4	6.7 ± 1.4

Lateral *s*′ (cm/s)	8.1 ± 1.3	9.0 ± 1.5	9.5 ± 1.7	10.1 ± 2.5	10.6 ± 1.9	11.6 ± 2.8
Lateral *e*′ (cm/s)	14.5 ± 2.2	16.4 ± 2.5	18.3 ± 2.9	18.2 ± 2.9	17.8 ± 3.1	18.1 ± 2.7
Lateral *a*′ (cm/s)	7.2 ± 1.8	7.1 ± 1.5	7.1 ± 1.9	6.7 ± 1.7	6.7 ± 1.8	6.5 ± 1.6
Lateral *e*′/*a*′ ratio	2.1 ± 0.5	2.4 ± 0.7	2.7 ± 0.7	2.8 ± 0.7	2.8 ± 0.8	2.9 ± 0.7
Lateral *E*/*e*′ ratio	6.8 ± 1.3	6.1 ± 1.4	5.4 ± 1.2	5.1 ± 1.1	5.3 ± 1.0	5.1 ± 1.0

Average *s*′ (cm/s)	7.8 ± 0.8	8.4 ± 1.1	8.5 ± 1.1	9.0 ± 1.6	9.3 ± 1.2	10.1 ± 1.9
Average *e*′ (cm/s)	13.3 ± 1.7	14.6 ± 1.8	15.7 ± 1.7	15.8 ± 2.0	15.8 ± 1.9	16.0 ± 2.1
Average *a*′ (cm/s)	7.2 ± 1.4	6.8 ± 1.2	6.7 ± 1.5	6.5 ± 1.4	6.3 ± 1.2	6.5 ± 1.4
Average *e*′/*a*′ ratio	1.9 ± 0.4	2.2 ± 0.5	2.4 ± 0.6	2.5 ± 0.6	2.6 ± 0.5	2.6 ± 0.5
Average *E*/*e*′ ratio	7.4 ± 1.2	6.8 ± 1.3	6.2 ± 1.2	5.8 ± 1.0	5.9 ± 0.9	5.8 ± 1.0

*a*′: peak late diastolic mitral annulus velocity; *E*: peak early diastolic transmitral flow velocity; *e*′: peak early diastolic mitral annulus velocity; *s*′: peak systolic mitral annulus velocity.

**Table 4:** Coefficients for regression equations relating left ventricular longitudinal indexes and age. For each index, application of the corresponding third-grade equation provides the expected logarithmic value according to age. In a patient with given age, this allows calculation of the *z* score that corresponds to the observed value of the index in that patient (see text).

	Age^3^	Age^2^	Age	Intercept	MSE	*R* ^2^
*s*′	8.3 · 10^−5^	−1.9 · 10^−3^	0.031	2.01	0.161	0.24
*e*′	2.6 · 10^−4^	−8.4 · 10^−3^	0.088	2.45	0.141	0.24
*a*′	2.8 · 10^−5^	2.3 · 10^−3^	−0.040	2.03	0.200	0.11
*E*/*e*′ ratio	−1.9 · 10^−4^	7.5 · 10^−3^	−0.097	2.15	0.198	0.25

*A*: peak late diastolic transmitral flow velocity; *a*′: peak late diastolic annulus velocity; *E*: peak early diastolic transmitral flow velocity; *e*′: peak early diastolic annulus velocity.

**Table 5:** Independent predictors of left ventricular longitudinal indexes in the study population, calculated as the average of septal and lateral values.

	*β* coefficient	*p* value
*s*′ (model *R* ^2^ = 24.1%, *p* < 0.0001)		
Age	0.491	<0.0001

*e*′ (model *R* ^2^ = 21.2%, *p* < 0.0001)		
Age	0.334	<0.0001
*E*/*A* ratio	0.207	<0.0001
Tricuspid *s*′	0.108	0.025

*a*′ (model *R* ^2^ = 16.0%, *p* < 0.0001)		
Heart rate	0.265	<0.0001
*E*/*A* ratio	−0.184	0.0007
Tricuspid *s*′	0.125	0.010

*E*/*e*′ ratio (model *R* ^2^ = 19.9%, *p* < 0.0001)		
Age	−0.369	<0.0001
Heart rate	0.108	0.077

*A*: peak late diastolic transmitral flow velocity; *a*′: peak late diastolic mitral annulus velocity; *E*: peak early diastolic transmitral flow velocity; *e*′: peak early diastolic mitral annulus velocity; *s*′: peak systolic mitral annulus velocity; tricuspid *s*′: peak systolic tricuspid annulus velocity.
